# K-12 Life Skills Education, Independence, and Employment of Autistic Individuals: Giving Voice to Autistic Adults

**DOI:** 10.1007/s10803-024-06304-7

**Published:** 2024-04-03

**Authors:** Jenna Christine Zatz, Judith R. Harrison

**Affiliations:** Department of Educational Psychology, Rutgers, The State University of New Jersey, New Brunswick, USA

**Keywords:** Autism spectrum disorder, Life skills, Autistic adults, Employment, Life skills instruction, Special education, Job sites

## Abstract

Autistic adults are often challenged to engage in and complete life skill tasks independently and are underrepresented in employment. No prior study has explored the perceptions of autistic individuals regarding K-12 life skills experiences and postsecondary employment. As such, the purposes of this study were to explore the association between components of life skills instruction and employment for 12 autistic individuals, and to elicit the perspectives of and experiences in K-12 education and employment of six autistic adults. As this was a mixed methods study, surveys and semi-structured interviews were conducted. Results of Fisher’s test indicated no statistically significant associations between employment and instructional components; however, the associations between employment and household chores [Cramer’s V = .60]; cooking [Cramer’s V = .66]; one-on-one instruction [Cramer’s V = .63]; and field trips [Cramer’s V = .41]) were large. The associations between employment and job site training [Cramer’s V = .33] and token boards [Cramer’s V = .33]) were moderate. Three themes and 10 subthemes emerged. Specifically, participants remembered Memorable Components from K-12 instruction: (a) job site training, (b) field trips, and (c) household chores. Participants perceived Beneficial Practices as: (a) job site training and (b) skills learned. Participants described shortcomings of K-12 instruction as need (a) for more skills training, (c) for social skills training, (d) to eliminate unnecessary instruction and (e) to carefully consider student placement. In conclusion, participants described experiences that helped them gain and attain post-secondary employment. More specific individualized programming in K-12 instruction would be beneficial to develop independence and post-secondary employment.

Throughout history and withstanding countless reforms, the inherent purpose of public education has been and continues to be to give children the tools, choices, and knowledge to function as contributing members of society under the reigns of state legislatures, district administrators, and educational professionals (Tyack & Cuban, 1995). For typically developing individuals, K-12 education usually meets this goal and leads to successful transitions to post-secondary life. However, even with the Individuals with Disabilities Education Improvement Act (IDEIA) mandating free and appropriate education, individualized education programs (IEPs), and least restrictive environment, autistic individuals are often left without the skills needed to engage independently in life, thus leaving many of them unemployed, and often disengaged from society (Carter et al., 2012).

The national rate of autism spectrum disorder (ASD) is 1 in 44 individuals (Data Statistics on Autism Spectrum Disorder, 2020). Autistic individuals frequently do not acquire skills at the same rate as their typically developing peers, specifically in communication, social skills, and motor skills (Autism Spectrum Disorder, 2013). The level of skill and need varies as they are a heterogeneous group with a limitless range of diagnostic presentations across developmental history, intelligence, comorbidity, and severity (Kucharczyk et al., 2015). For example, one autistic individual might write and read on grade level, but struggle with social skills and another might have a cognitive disability and be non-vocal. As such, individual post-secondary goals (e.g., employment, post-secondary education) are likely to be very different for individuals with different support needs. As such, one common means of categorizing autistic individuals is by levels of severity identified by needed support (i.e., Level 1-Requiring Support, Level 2-Requiring Substantial Support, Level 3-Requiring Very Substantial Support (American Psychological Association; Autism Spectrum Disorder, 2013).

The focus of the current study was on employment after high school as employment rates for autistic individuals are disproportionately low and appear to decline over time with minimal increases in independence and some regression (Taylor et al., 2015; Taylor & Malick, 2014; Taylor & Seltzer, 2011). According to Roux et al. (2017) only 32% of autistic individuals attained employment within the first 2 years after high school. This is unfortunate as employment is vital to the well-being of most individuals, as it is related to developing independence, quality of life, and positive mental health (Flint et al., 2013; Grun et al., 2010). Additionally, many autistic individuals have a strong desire to work and have a right to the opportunity to be productive and contributing members of society (Anderson et al., 2020). As such, understanding the relationship between K-12 life skills instruction and employment is essential.

## Prerequisite Life Skills for Employment

Skill deficits potentially contributing to the high rates of unemployment of autistic individuals are couched in the category of life skills, specifically in the areas of personal hygiene, communication, social, pre-vocational, and employment skills (Gushanas & Thompson, 2019; Kodak & Bergmann, 2020). Some autistic individuals struggle to independently engage in daily living skills, thus potentially decreasing the probability of employment (Taylor & Seltzer, 2011; Taylor et al., 2015). Additionally, when autistic individuals were queried about postsecondary challenges, they reported the most challenging aspects were social skills (e.g., reading/responding to social cues), communication, pre-employment skills (i.e., resume writing), and mental health (feelings of anxiety; Anderson et al., 2020; Angulo-Jiménez & DeThorne, 2019; Black et al., 2019; Hedley et al., 2017). Therefore, it is likely that developing life skills is beneficial for positive employment outcomes of autistic individuals.

## Life Skills Instruction

Two types of curricula are used to guide K-12 instruction (i.e., academic or standards-based instruction, functional/life skills instruction; Bouck, 2012; Chaing et al., 2017), and several researchers have explored the connection between curricula choice and employment status. However, the significance of the type of curriculum used in K-12 instruction and employment outcome remains unclear. When conducting secondary analyses with data from the longitudinal NLTS-2 (Newman et al., 2011), in which 5,000 autistic individuals and their parents were surveyed or interviewed about secondary and post-secondary experiences, including curricula, studies revealed mixed results. One study (Chaing et al., 2017) found that students who received life skills instruction in high school were more likely to independently engage in skills such as counting change, reading and comprehending community signs, making a small meal, and higher competency in social skills. However, two studies (Bouck, 2012; Bouck & Joshi, 2015) found that the type of curricula had no bearing on post-secondary outcomes. It is possible that these mixed findings are related to the heterogeneity of the autistic individuals or that curricula were considered without consideration of individual instructional components.

## IDEAL Model Components

Within life skills curricula, practitioners often implement components of different frameworks (e.g., Applied Behavior Analysis, Treatment and Education of Autistic and Related Communication Handicapped Children) to teach autistic individuals (Callahan et al., 2008, 2010). From those approaches, parents of autistic individuals, administrators, and teachers identified valuable components that fell within five specific areas: (a) individualized programming, (b) data collection, (c) use of empirically based strategies, (d) active collaboration, and (e) focus on long-term outcomes (i.e., IDEAL components). Within these five areas, several elements are particularly relevant to independence and employment (see Table 1). For example, several studies (Bouck, 2012; Carter et al., 2012) found that community-based instruction was beneficial, as it provided opportunities for autistic individuals to practice and generalize previously acquired skills on job sites and in community and in real-life settings. Additionally, researchers (Bennett et al., 2013; Berezenak et al., 2012; Strickland et al., 2013) found that video modeling, an empirically based strategy, resulted in increased independence and ability to demonstrate pre-vocational skills, such as t-shirt folding, clerical skills, and photocopying.

**Table 1:** IDEAL components and sample of relevant elements of IDEAL model components

IDEAL model component	Sample of relevant elements of component
Individualized programming	· Individualized programming which addresses developmental content areas (communication, social development, adaptive behavior, cognitive development, and adaptive physical education) · Individualized programming which addresses non-academic content areas (attending to elements of the environment, language comprehension, use of language, social skills, self-help skills; household chores; cooking) · Instructional programming that targets all affected areas of development in order to meet the unique characteristics and individualized needs of each child (e.g., completing chores)
Data collection	· Use of curriculum-based assessments to make data based instructional decisions · Use of valid and reliable assessment instruments to conduct pre- and post-tests/determine student progress toward benchmarks · Ongoing monitoring and evaluation of the program · The measurement, documentation of student progress toward annual goals/objectives and reporting such data to parents
Empirically demonstrated strategies & interventions	· The use of specialized curricula and strategies to teach communication skills (e.g. script fading, written social phrases, functional communication training, and teaching spontaneous self-initiated responses/verbal imitations; token economy) · Use of reinforcement (e.g., praise; positive feedback) · Use of task analyses to systematically teach new skills · The use of incidental teaching and/or naturalistic teaching · Active engagement of students throughout the day in intensive instructional programming with repetition of skills to promote practice
Active collaboration	· Direct involvement of parents and family members · Peer-mediated interventions, including the involvement of typically developing peers in collaborative · Activities, including social skill training groups and play groups
Long-term outcomes	· Emphasis on life skills, and vocational/employment/occupational skills training through school-based and community/work-based learning experiences (e.g., social skills; job site, field trips) · Transition programming at all ages and levels · Intensive generalization programming across different materials and environments (e.g., job sites) · Use of different materials, interventions, and environments to promote generalization (e.g., job sites)

Components and elements are adapted from Callahan et al. (2010)

In sum, although results were mixed regarding the relationship between functional curricula and employment status, emerging studies provide preliminary evidence of the importance of the IDEAL components of functional curricula. However, no prior studies have directly given autistic adults the opportunity to share their experiences in K-12 life skills instruction and their perception of the association between components of secondary instruction, life skills independence, and employment status. This is unfortunate as amplifying the voices of autistic adults has the potential to reveal the most authentic viewpoint and firsthand recollections of life skills education, employment experiences, and their relation to one another. As such, the purpose of the current study was to explore the experiences of K-12 programs as perceived by autistic individuals and the relationship with independence and employment. This study was guided by the following research questions.

1. What is the association between IDEAL components of instruction and employment status based on autistic individuals’ reports?
2. What are the lived experiences of autistic individuals in K-12 instructional programs and what are the perceptions of the benefits or shortcomings?

## Method

### Design

This mixed method study was conducted in two phases, Phase 1 (survey) and Phase 2 (interviews).

### Participants

#### Phase 1: Survey

A sample of 12 autistic individuals were included in the survey if they (a) had a diagnosis of ASD; (b) were of legal employment age (16 years old minimum); (c) were receiving or had received life skills instruction in a public or private school setting; and (d) were able to independently communicate thoughts, feelings, and experiences. See Table 2 for details of the final sample. Nine individuals required support (level 1); two individuals required substantial support (level 2) and one individual required very substantial support (level 3). Six participants were male and six were female. A majority of participants were white (*n* = 10) and two were Latinx. Seven participants reported they had 8 plus teachers in one day indicating they were not in a self-contained classroom setting. Four individuals reported they had two to three teachers in a day suggesting they had likely been in a self-contained classroom setting. From these 12 individuals, a subsample of six autistic individuals were interviewed.

**Table 2:** Survey participant characteristics

	Emily	Tim	Rob	Ally	John	Rebecca	7	8	9	10	11	12
ASD level*	3	1	1	1	2	1	1	1	1	2	1	1
Gender	F	M	M	F	M	F	M	F	F	M	F	M
Race	C	C	C	L	C	C	C	L	C	C	C	C
# of Teachers senior or current year	2–3	8+	8+	2–3	8+	8+	8+	1	8+	2–3	8+	2–3
Employed at time of survey	Y	N	N	Y	Y	Y	Y	Y	S	Y	S	Y

*Level 1 = individual requires support; level 2 = individual requires substantial support; level 3 individual requires very substantial support

*Y* yes, *N* no, *C* Caucasian, *L* latinx, *S* still attending classes

#### Phase 2: Interviews

A total of six participants volunteered for interviews (see Table 3 for participant details). Final participants follow: (1) Emily^1^ was a 22-year-old female who graduated from a public high school and was employed at Cookies for the Community (pseudonym) at the time of the study. (2) Tim was a 22-year-old male who attended two public high schools and was unemployed at the time of the interview but worked at Bob’s Store for 2 years prior to the pandemic. (3) Rob was a 34-year-old male who graduated from a public high school and was unemployed; however, he had been employed at Windmilltown for the Student After Care (SAC) for over 14 years prior to the pandemic. (4) Ally was a 22-year-old female who graduated from a public high school and was recently employed at MJ Minimum at the time of the interview. (5) John was a 37-year-old male who graduated from high school in Nebraska and was employed at Right Grocery. (6) Rebecca was a 27-year-old female who graduated from a therapeutic out of district high school, after leaving a public high school her sophomore year. At the time of the interview, Rebecca was employed at BeverageWay beverage company.

**Table 3:** Interview participant information

	Emily	Tim	Rob	Ally	John	Rebecca
ASD level*	3	1	1	1	2	1
Gender	F	M	M	F	M	F
Race	C	C	C	L	C	C
Main type of high school classroom**	Self-contained	Inclusion	Inclusion***	Self-contained	Inclusion	Inclusion (1st), self-contained (2nd)
Employed at time of interview	Y	N	N	Y	Y	Y

*Level 1 = individual requires support; level 2 = individual requires substantial support; level 3 individual requires very substantial support

*Self-contained* received life skills instruction in a self-contained setting, *Inclusion* general education with general and special education teachers or paraprofessional; *Y* yes, *N* no, *C* Caucasian, *L* latinx

### Data Collection Instruments

#### Phase 1: Surveys

The final individual survey included 25 questions, 14 dichotomous (yes/no) questions, eight multiple choice and three checklists. Six questions queried independence level; one question queried current leisure activities; four employment status; one employment responsibilities; and 13 K-12 experiences. Survey questions were short (i.e., 10 words maximum per question) and utilized familiar phrases likely to be heard at school and everyday life to promote independent comprehension of the questions. Multiple choice questions and checklist questions were limited to a maximum of four answer choices.

The survey was developed by modifying questions and responses from the National Longitudinal Transition Study-2 (NLTS-2) survey. The NLTS-2 survey is parent and student assessment used in the NLTS-2 Longitudinal Study (Newman et al., 2011) that queries individuals' experiences with life skills training, social issues, and post-secondary experiences (Bouck, 2012; Carter et al., 2012).

We selected questions from the NLTS-2 (waves 1–5) and New Jersey Comprehensive Assessment Tool (NJCAT) that targeted employment status (NLTS-2), level of independence (NLTS-2; NJCAT), and K-12 experiences (NLTS-2). Specifically, questions were included on the survey if they queried participants about: (a) independence level; (b) current leisure activities; (c) employment status; (d) employment responsibilities; and (e) K-12 experiences. In regard to K-12 experience, we specifically asked about (a) instructional strategies as IDEAL components, (b) skills practice, and (c) IEP involvement. The goal was to align with the purpose of this study and the literature on developing independence in life skills, IDEAL components, and employment. Questions were modified from Likert Scale type questions to multiple choice and checklist type answer responses. Reliability with all 12 items was questionable (ɑ = .65), so we removed one item as it had a negative correlation with the total score (Tavacol & Dennick, 2011) increasing the reliability to acceptable (ɑ = .73).

#### Phase 2: Interview Protocols

A total of 22 open ended questions were included on the interview protocol. Questions were modified from the NLTS-2 interview protocol and expanded upon. The protocol included interview questions from waves two through five. The NLTS-2 protocols were modified to be semi-structured. Specifically, each new question allowed participants to elaborate, which led to a naturally flowing discussion as opposed to a question-and-response format.

Overall, we selected questions that asked about (a) independence level; (b) current leisure activities; (c) employment status; (d) employment responsibilities; and (e) K-12 experiences. In regard to K-12 experience, we specifically asked about (a) elements of IDEAL components (b) skills practice, and (c) IEP involvement. Specifically, we included questions that elicited components that were utilized in K-12 life skills instruction, including community trips, vocational training, types of employment, job site settings, and satisfaction or dissatisfaction with K-12 life-skills education.

To individualize for unique needs, the semi-structured interview protocol included a range of questions as well as individualized and differentiated probes based on cognitive ability level. As cognitive levels varied, probing was carefully crafted with relatable wording and language used in classroom settings and was tailored to the individuals’ recollections and needs at that moment. The length of questions was short to promote independent comprehension. The first portion of the protocol sought to elicit memories of life skills, the second portion sought to elicit memories related to the employment process, and the third portion sought to elicit evaluations and perceptions of their life skills education as they relate to employment status.

### Procedures

Procedures are described by phase and categorized as (a) recruitment and (b) implementation.

#### Phase 1: Survey

First, the researchers emailed a flyer (with contact information) and telephoned seven day-programs and organizations throughout a state in the Northeastern United States including Center for Adult Autism Services (RCAAS), Eden, the ARC, Autism Speaks, Easter Seals, Cookies for the Community, and Disability Allies. Additionally, the researcher shared the flyer on social media post. Potential participants contacted the researcher if interested.

Surveys were administered via Qualtrics with the link accessed through either scanning a QR code or clicking a link embedded in both the email and social media post. Survey questions were delivered one at a time and all questions were required to be answered prior to moving on. Survey questions were structured to provide insight into classroom setting, independence level, and communication level through multiple choice and checkbox responses (e.g., how many students are/were in most of your classes; how many teachers did you have senior year or current year?). A second survey was conducted with parents and results will be reported in a separate manuscript published in another study that provided information as to their child’s level within the autism spectrum.

#### Phase 2: Interview

The survey prompted individuals to indicate whether they would be willing to participate in an interview. When survey respondents indicated interest in participating in the interview, an interview was scheduled at a time and place that was convenient for the participants (two in-home and four via Zoom).

Interviews were conducted in person and via zoom by the first author. All interviews were conducted by the first author. Two individuals opted for in-person interviews. As such, the first author went to the participants’ homes and four individuals opted to conduct their interview via Zoom. Interviews with individuals were conducted without parents to (a) ensure participants were comfortable speaking freely and (b) eliminate potential prompting or subjectivity stemming from parent input. When parents remained nearby, the researchers kindly and consistently reminded parents to refrain from prompting, which was respected by each parent. Throughout interviews, ample wait time was given to allow the individual to process the question. Each interview was audio recorded. Each of the six autistic individuals communicated with varying degrees of spoken language ranging from full sentences with details (i.e., Rob, Rebecca, Ally) to short phrases (i.e., Emily, John, Tim).

A second interview was conducted with parents and results will be reported in a separate manuscript; however, we included parent report of the individual's level of need within the autism spectrum.

### Data Analysis

#### Quantitative Analysis

To evaluate the association between individual report of elements of life skills instruction on the survey in Phase 1 and employment (employed/unemployed) and the association between parents’ report of elements of life skills instruction and employment in Phase 1, we conducted a Fisher’s Exact Tests (FET) with five questions (individual) that directly queried elements of life skills instruction and employment status. We elected to use Fisher’s Exact Test as our sample size was small with a few cell counts less than five (McDonald, 2022). Fisher tests were used to determine the association between life skills instruction components as reported by individuals on the survey in Phase 1 and employment status (employed/unemployed) due to small sample size (McDonald, 2022). Due to insufficient power the statistical significance of our results, they are considered preliminary; however, we calculated Cramer’s *V* was to determine the magnitude of association not impacted by sample size (Foster et al., 2022).

#### Qualitative Analysis

To explore the lived experiences of autistic individuals in life skills instruction programs and their perceptions of their K-12 life skills training, five stages of qualitative analysis were conducted following the procedures of Creswell and Creswell (2018). First, audio recorded interviews were transcribed by hand. Second, the two researchers reviewed the transcribed interviews thoroughly. Third, the researchers read the transcripts multiple times (i.e., 3 times). Fourth, the two researchers collaboratively coded the data using Dedoose® (Version 9.0.17 2021). Fifth, themes were created based on similarities, parallels, and trends within the codes.

Through a simultaneous combination of deductive and inductive coding (Creswell & Creswell, 2018), iterations of codes were developed across three rounds of coding using Dedoose®. The researchers began by identifying and defining three broad priori codes (i.e., life skills instruction, community-based instruction, and vocational skills) from the work of Bouck (2012), Callahan et al. (2010), Carter et al. (2012), and Chaing et al. (2017; See Table 4). We began by identifying excerpts in the transcripts that met the definitions/descriptions on Table 4.

**Table 4:** Deductive and inductive codes

Code	Definition/description	Examples	Deductive	Inductive
Life skills instruction	Direct instruction or active practice in personal hygiene, household chores, social skills, pre-vocational skills	Doing laundry Cooking Brushing teeth	x	
Community-based instruction	Instruction led by K-12 teachers outside of the classroom and/or school setting (e.g. grocery stores)	Field trips	x	
Pre-vocational/vocational skills	Skills, behaviors, and emotions needed to get a job and remain engaged in the job	Resume writing	x	
Personal hygiene	Tasks involving personal cleanliness and bodily health	Brushing teeth		x
Pre-employment skills	Skills needed to establish a foundation for independence in applying for jobs	Interview skills		x
Social skills	Effective and appropriate expressive and receptive communication skills	Asking follow up questions		x
Unnecessary instruction	Subject areas and/or content perceived as not needed for independence in employment	Some academic content		x
Job site training	Direct instruction and engagement in employment tasks at potential employment settings within the community	Working at a store as part of school program		x
Benefits	Aspects of life skills education reported as being helpful in attaining and/or maintaining employment	Direct practice of life skills		x
Shortcomings	Aspects of life skills education reported as being unhelpful in attaining and/or maintaining employment and/or recommendations to improve life skills education as it relates to employment	Incorrect student placements		x

Simultaneously we engaged in inductive coding, in which we developed codes within and between transcripts through observation and reflection (Saldaña, 2021) using a combination of descriptive (very brief summary-one word), structural (labeled structural aspects, such as where what who how) and values coding (reflects participants beliefs, feelings) reviewing all transcripts. In the next iteration of coding new themes emerged. Across all iterations with the use of both deductive and inductive coding, eight code categories (i.e., life skills instruction, personal hygiene, pre-employment skills, social skills, unnecessary instruction, job site training, benefits, shortcoming) emerged. We should note that codes evolved across iterations with the final codes in Table 4. Next, themes were created from the codes by reflecting on patterns and trends in the data and drawing meaning from the codes to answer the research questions. Specifically, we grouped codes into themes by organizing them into coherent and consistent accounts (Braun & Clark, 2006) to create broad themes and subthemes.

### Reliability and Validity

Reliability of phase 1, survey data was measured through the calculation of Cronbach’s alpha. Validity of phase 2, qualitative interviews was established through three procedures. The first validity strategy was triangulation. The researchers compared codes across all interviews and if a code was found across more than one participant’s experience, it was kept (Creswell & Creswell, 2018). The triangulation of data occurred by cross-analyzing codes across interview responses and researchers across the development and use of both inductive and deductive (based on prior literature) codes. Three rounds of coding were completed by the first author to finalize the codes. The two authors discussed the coding in weekly meetings. To promote intercoder reliability we followed recommendations outlined by O’Connor et al. (2020) which included consistent reflexive and reflective dialogue occurred between the two researchers during all rounds of coding. We consistently agreed on codes indicating that codes were clearly and objectively defined for deductive coding and similar trends were found from the transcripts (O’Connor and Joffe, 2020).

Second, we conducted member checking to ensure the accuracy of findings by sharing semi-polished results of data analysis and seeking feedback from individuals (Creswell & Creswell, 2018) to ensure we captured their viewpoint correctly. We sought participant feedback by emailing participants typed transcripts in a secure email and asked participants to read the transcripts and use suggesting mode to make any comments. We also offered additional time to meet to discuss their feedback or to allow participants the opportunity to give verbal feedback. However, no such requests were made. Third, the researchers maintained an audit trail, a detailed record of procedures and decisions that were made (Creswell & Creswell, 2018). Throughout the current study, a detailed research journal was kept that included decisions made through the data collection and analysis processes. By following these strategies, every effort was made to maintain trustworthiness, authenticity, and credibility (Creswell & Creswell, 2018).

Furthermore, we were consistently aware of our experiences, beliefs, and potential biases. The first author was a teacher of children with ASD at the time of the study and the second was previously a teacher of students with disabilities, leading to established opinions and beliefs. As such, we did not include students who we had taught to maintain a neutral positionality. Additionally, throughout the validity procedures described above, we consistently were transparent with each other regarding our potential biases and made every effort to remove those biases from our analysis of individuals’ voices by adhering to the transcripts.

## Findings/Results

In the following sections, we report the results of the first research question (association between components and employment; see Table 5). We report statistically significant findings and effect sizes, with a reminder that statistically significant results were interpreted as preliminary due to the sample size. Second, we report the result of the second research question (lived experiences) with data analyzed qualitatively for individuals (see Table 6).

**Table 5:** Associations between life skills instruction components and employment status

Component	Unemployed	Employed	Cramer’s *V*	χ^2^	*p* value
Practicing household chores (no)	2	3	.60	4.29	.12
Practicing household chores (yes)	0	5			
Practicing cooking (no)	2	2	.66	4.83	.09
Practicing cooking (yes)	0	5			
Job site training (no)	0	2	.33	1.33	.51
Job site training (yes)	2	6			
Token boards (no)	1	5	.33	1.31	.52
Token boards (yes)	1	3			
One-on-one instruction (no)	1	0	.63	4.8	.09
One-on-one instruction (yes)	1	8			
Community field trips (no)	1	1	.41	2.0	.37
Community field trips (yes)	1	7

**Table 6:** Research question and related themes

Research questions	Themes
What are the lived experiences of autistic individuals in K-12 instructional programs and what are the perceptions of the benefits or shortcomings?	· Memorable components · Beneficial practices: skill to confidence · Job site training · Skills learned · Shortcomings: more and less · Need for more Skills training · Need for social skills training · Unnecessary instruction · Student placement

### Phase 1: Survey

No statistically significant associations were found between employment status (employed/unemployed) and any life skills instruction component (See Table 5). However, the association between four components and employment status had large effects (practicing household chores [Cramer’s *V* = .60]; practicing cooking [Cramer’s *V* = .66]; one-on-one instruction [Cramer’s *V* = .63]; community field trips [Cramer’s *V* = .41]), and two had medium effects (job site training [Cramer’s *V* = .33]; token boards [Cramer’s *V* = .33]).

### Phase 2: Interviews

Three themes and six subthemes emerged to identify individuals' perceptions and recollections of their experiences in K-12 Life Skills. Individuals excitedly and, at times briefly, described their experiences in high school. The researcher provided clarifying information as needed, but ultimately individuals understood and provided very valuable information.

#### Memorable Components

The first theme that emerged was Memorable Components. Individuals frequently simply told us what they remembered without elaboration; however, at times, more explanation was included in their answers. Across individuals five memorable components of life skills instruction were discussed: (a) job site training, (b) practicing household chores, (c) participating in community field trips, (d) practicing personal hygiene, and (e) practicing pre-employment/job skills (See Table 7).

**Table 7:** Memorable components recalled

Component/aspect	Number of individuals reporting
Job sites	6
Household chores (e.g., baking, laundry, cooking small snacks, vacuuming, making the bed)	4
Community field trips	4
Pre-employment (e.g., resume writing, interview practice)	3
Personal hygiene (e.g., brushing teeth, washing face, combing hair)	2

##### Job Site Training

Participants were excited to tell the researcher about their experiences with job site training, which involved practice at a job (free of pay) at community businesses. All individuals remembered working at a job site, some described multiple sites that allowed them to practice skills, such as scanning books, delivering mail, clerical work, and custodial tasks. Emily reported working at many job sites, such as Cookies for the Community, TrioShine, the West Brownstone Public Library, and the West Brownstone Administration Building. At each job site, Emily recalled practicing different skills including scanning at the library and delivering mail at the administration building. Tim reported that his only job site was Bob’s Clothing Store, which led to a paying job at Bob’s post-graduation. Rob recalled working at Bullseye (a general merchandise retailer), as a “custodian and a cart attendant” and John recalled that his job site “was at HyVee…A grocery store” where he recalled being “a bagger back then.”

##### Household Chores

Individuals also remembered practicing household chores, such as cooking, making the bed, doing laundry, cleaning, and vacuuming. Tim and John both recalled learning to cook; specifically, “little snacks” as reported by Tim. Rebecca recalled, “We did mainly baking like sometimes we would make sweets…chocolate lollipops all that fun stuff.”

##### Community Field Trips

Participants also described their experiences on field trips, such as grocery shopping, visiting the public library, and a trip to an Orchard. Specifically, Rebecca, John, Tim, and Emily recalled opportunities to go on field trips into the community during the school day. Rebecca recalled, “We went to Price Co to pick things up for shopping. That was really fun. We would go to like Price Co. It was mostly Price Co. But there was a Grocery Rite we went…there.” John reported going on field trips to museums and the public library. Tim smiled as he recalled going on a community field trip “[The school] actually took us to Scrumptious Farms and then they actually took us to the actual orchard.” His comments seemed to emphasize the importance, but the unlikely event, of visiting exciting places.

##### Pre-employment Skills and Personal Hygiene

Fewer students talked about being taught pre-employment skills and personal hygiene than other components. Ally, Tim, and Rebecca remembered practicing pre-employment and job skills during life skills instruction, and all three reported developing a resume. Ally recalled practicing applying for jobs, which she felt helped her attain a job at Grocery Rite after graduating. Tim also reported practicing mock interviews at school. Rebecca reported she learned “how to do a lot of sorting and how to be polite and just like making resumes” at school. Two females reported practicing personal hygiene during life skills instruction. Emily said, “[I] practiced personal hygiene which included, washing hands and [going] to the bathroom and “[brushing] my hair.”

#### Beneficial Practices: Skills to Confidence

The second theme that emerged was Beneficial Practices: Skills to Confidence. Participants described practice of life skills during K-12 education that increased their confidence. Individuals varied in their perceptions of what was most helpful and two sub-themes (Job Site Training, Skills Learned) emerged; however, the common theme running through both was learning skills and gaining confidence.

##### Job Site Training

Five of the six individuals emphasized the importance of training on job sites, as highly beneficial for learning skills, building confidence, and future employment. On job sites individuals were given the opportunity to learn and practice skills required for future employment. For example, Rebecca described practicing skills at multiple job sites while she was in her Junior and Senior years of high school. She worked at a thrift store, a museum, and a restaurant where she practiced clerical work, and most importantly, this practice led to confidence and developing independence, which “is one of the reasons why [she] got [her] job…” Rebecca pridefully described her job responsibilities when she said, “I get to do all my fancy little clerical work” including using Excel. Rebecca elaborated on her job duties by explaining, “I get to post receipts, enter little documents. And sometimes I’ll sort things and put them away, scan and attach items, all good stuff.” This generalization from instruction to employment was not unique to individuals who needed less support, such as Rebecca. Emily, who was limited in verbal expression, reported that job sites helped her in performing her current job where she learned to label bags. When she graduated, she began working for the same company doing the same task.

Job site training gave individuals the opportunity to learn personal skills that helped them attain and maintain jobs, which required more than specific skills, such as filing and putting labels on products. Individuals must be able to engage in personal hygiene and demonstrate appropriate attitudes. As Rob reported, he learned, “Well [how] to be clean and be active and be like energetic. Make sure to get the job done when you’re in the environment.” He further explained that knowledge regarding how to show up to work on time and in a presentable way (e.g., clean, well groomed, dressed appropriately) were fostered during his life skills instruction. The skills he acquired were essential for making positive first impressions and maintaining positive relationships with others.

##### Skills Learned

All individuals described learning specific skills, or wishing they had, that directly related to developing independence and attaining and maintaining employment. Rebecca, Rob, and Ally emphasized the importance of pre-employment and vocational skills. Rob recalled “The life skills at school helped me to be prepared and to show us how to do those things like going through interviews, how to work on jobs, how to greet people, and like it’s like life. That’s how life goes.” Ally reported that elements including “[socializing], [receiving] instructions,” making resumes, practicing applying for jobs online, and the fact that she “learned time management” helped her with her job. Rebecca went a step farther and explained that learning how to perform skills needed for job tasks was more beneficial than learning about abstract academic concepts:

I feel like I actually learned [at] [the second school] compared to [the first school] where it was kind of just like mush. [That’s] the best way to describe it. Mom would say it was a very good educative experience in [the first school] and while that's true, when are you ever going to use math and history in normal jobs you get hired for? Like, in [the second school], I learned how to do a lot of sorting and how to be polite and just like making resumes. Like that seemed a lot more important, shouldn’t that be, we be, learning that more often?

Skills used throughout daily life and during employment were the most valued to autistic individuals. Skills related to post-secondary life, as opposed to academic skills (e.g., balancing equations, book reports, historical lessons), are most beneficial in employment.

#### Shortcomings

In addition to themes related to skills students learned and found valuable, four subthemes (i.e., Need for More Skill Training, Need for Social Skills Training; Unnecessary Instruction, and Student Placement) emerged as shortcomings, the training and opportunity that participants reported needing and not getting or getting and not needing.

##### Need for More Skills Training

Three individuals discussed pre-employment skills that would have been more helpful than other content they were taught. Some described skills that were necessary for their jobs, but not typically found in life skills curriculum. For example, Rebecca wished she had learned more computer skills, “I think making certain spreadsheets would have helped like the [spreadsheet] thing. I learned it with [a job coach] after high school.”

Others described the need for training at multiple job sites. Ally, who worked at a clothing store, explained that more exposure to different job sites would have been helpful, “… cause during the job sites I went to [in high school], I worked not at all at a clothing store”, where she went to work after high school. Still others wanted to learn more skills to get a job. Tim reported “…like how to get a job. Like what is needed, the steps. Like the resume and things like that” and about financial literacy. Ally reported it would have been helpful to “learn how to do taxes. I don’t do taxes now, but I will eventually. I have poor money skills.” Similarly, Rebecca reported it would have been helpful to learn “the taxing and billing nonsense like that. And even tips. Tips took a while for me to figure out, I still struggle with that.”

Clearly, there is an overall need for more opportunities to practice skills directly related to post-secondary life. As mentioned previously, skills most related to employment and post-secondary life are valued as most beneficial to autistic individuals. As such, it makes sense that these would be the skills individuals would like educators to focus on in K-12 instruction.

##### Need for Social Skills Training

The Need for Social Skills Training emerged as a subtheme. Half of the participants recognized and emphasized their struggles with social skills and expressed desire for more focused instruction, specifically in communication and socialization.

When discussing social skill building activities they wished would have been incorporated, participants described more instruction and practice in “basically like [getting] to know each other and like learn how to keep conversations going (Tim).” John felt his life skills program should have provided a balance between social skills and job skills. Without that, he felt like he did not have the skills he needed to be successful. He reported that he wished he had more “new people” in life skills instruction to practice “conversations.” He explained that the most challenging part of getting his job at the grocery store was “meeting new people.”

##### Unnecessary Instruction

In addition to describing skills that they would have liked to have been taught, two participants were clear about Unnecessary Instruction or elements of life skills that were perceived as not necessary for functioning in daily life and employment. Rebecca was animated as she described a class that she perceived as taught below her level and with unrealistic examples.

Oh my gosh I have to tell you about overcoming obstacles. This was a class that I think was meant to make people do problem-solving, but it was very childish problem-solving. Very tiny stuff that would probably never, but an actual situation not like something involving tiny stuff that would probably never, but an actual situation not like something involving anxiety issues or like emotionally overwhelmed or depression. No no, it was just like little hypotheticals. Like nonsense that you would see in an Ad-Lib book or whatever. And like eventually I think everyone stopped taking this class seriously. And like, I would have an aide attend with me every class and eventually we were just like let's go let's get out of here. This is nonsense. And all the students were misbehaving. And the teacher, and someone eventually decided to pull a prank on the teacher when I wasn’t there by placing their shoes by the window and then hiding in the closet and so the teacher would scream because she thought the student jumped out the window. So that was a disaster, and that class did not last very long.

It was clear that Rebecca did not value unrealistic, hypothetical situations.

Other participants described the need for drivers’ education as a component of Life Skills. Ally reported that it would have been helpful to learn, “…Uhm like learning how to drive and stuff. In high school yeah, I took like the learners permit like the learning test like the written test and stuff, but it’s not like they actually showed me how to try to drive.” In sum, more time should be placed on teaching the skills that are perceived as beneficial to individuals, and are directly tied to fostering independence, daily living, and employment.

##### Student Placement

The last theme that emerged was Student Placement across three participants. This theme is defined as student placement that resulted in negative experiences for autistic individuals in high school resulting in minimal help with independence and skill needed for employment. In fact, some participants felt their self-esteem/self-worth was damaged because they were placed in self-contained settings with students who needed more support. Ally described her self-contained classroom as being “pretty much just filled with special needs kids like ones that are more special than I am.” Ally elaborated:

It was, like, uncomfortable and I felt like I was being treated like I was being treated like one of those kids so I felt more stupid and stuff I guess. Like if they talked to me like a normal person I probably wouldn’t have like I guess I just felt like more of a dumbass than I actually am at school stuff.

Self-esteem was also in question in general education settings as participants explained that they were treated poorly in these “inclusive” settings. Rob reported that not all the “like 20 kids” in his inclusion class were kind. “Some of them were nice and some of them thought that I was, like, different. Like I didn’t know when I had this condition. My mom told me that like after school and um I just didn’t know why because I was just being myself.” Rebecca explained conflicts with other students in her inclusion classes:

Lots of kids that would lie to me and that's where I started forming trust issues. Like [Mom] was complaining about, Mom mentioned that I wasn’t very social. There was a reason for that, it’s that people were being kind of terrible. She conveniently leaves that out. I just started withdrawing myself because unfortunately people like to use me.

All these situations left individuals doubting their self-worth, diminishing their self-esteem, and questioning their ability to independently complete job tasks. Individuals spend countless hours in K-12 instruction, so instead of creating a negative environment between students, thoughtful student placement can prevent a toxic climate and promote positive self-esteem and confidence.

### Summary of Findings

Overall, results across individuals indicated that practicing skills learned at job sites in K-12 education was the most beneficial in obtaining employment after graduation. Additionally, spending time practicing specific skills including household chores, personal hygiene, and pre-employment skills was highly beneficial in K-12 life skills instruction. This time spent practicing these skills during K-12 life skills instruction promoted independence and was therefore linked to employment following high school graduation.

Results also revealed many shortcomings. Specifically, participants felt that more time to learn and physically practice necessary life and social skills in authentic situations. Furthermore, it was clear that appropriate placement was essential for learning and demonstration to occur.

## Discussion

Although the findings from surveys and brief interviews have been conducted in the literature regarding best practice to develop independence and increase employment of autistic individuals, this is one of the first studies to provide space for uninterrupted and unfiltered voices of individuals, those who have directly experienced K-12 instruction as an autistic individual. It was not our intent to explore specific experiences with a particular curriculum, but instead to give autistic individuals an opportunity to reflect on their experiences and different components of instruction that they remembered and wanted to discuss. Individuals described both beneficial practices and shortcomings. Prior to further discussion, we caution readers to interpret the results and discussion as preliminary as our sample was small. Furthermore, we encourage readers to interpret the results in relation to the subsample of autistic individuals in the current study who received life skills instruction in secondary schools and who went directly into employment after high school.

Overall, we found that participants valued learning and practicing real-life skills in authentic settings in both the surveys and interviews. Individuals in this sample valued their experiences at job sites and in the community and described a need for more of those experiences. Five out of six individuals reported job skills as very helpful, more time on job sites as an activity that would have been more helpful, at times, about the amount of time spent in instruction not directly related to their perceived futures. Although without fluffy terminology and jargon that we often use as educators, participants expressed the value of components of functional curricula, especially those considered IDEAL components within opportunities to engage in direct practice of specific life skills in both classroom and job site settings. Many participants asserted that these components of functional curricula led to the development of independence and successful employment. Engagement in these aspects were positively associated with successful employment status. Furthermore, individuals described a need for improvement in K-12 life skills, pre-vocational, and vocational instruction, especially in creating more opportunities to practice specific skills and to ensure that placement and instruction is truly individualized to meet their long-term needs. We believe K-12 education should meet the needs of learners, not the needs of members of state legislatures, district administrators, and professionals within the educational field.

As we discussed in the introduction, the heterogeneity of autistic individuals cannot be overstated. Along with characteristics of individuals, postsecondary goals are very diverse. It is likely that the findings here related to the importance of a functional curriculum were associated with the goals and characteristics of the autistic individuals in this study. It is important to note that every autistic individual included in the study received life skills instruction during the academic day and became employed immediately after high school. As such, their focus was on life skills instruction.

Simply exploring the components of instruction that individuals remembered and giving them the opportunity to extrapolate provided preliminary insight into what the autistic individuals in the current study valued. It quickly became clear that, similar to the findings of Bouck (2012) and Carter et al. (2012) who used data from interviews of educators and parents, participants valued instruction in community-based settings (i.e., job sites), a component of functional curriculum and an IDEAL component. These opportunities provided individuals time to practice and generalize previously acquired skills in natural settings (e.g., job sites, stores, restaurants).

Similarly, autistic individuals in the current study described the benefit of practicing skills often utilized in the employment process (i.e., pre-employment skills), such as resume writing. Across the survey and interview, individuals focused on explicit instruction and the practice of skills needed to develop independence and attain employment taught through IDEAL components of functional curricula. Lack of adequate skills training in pre-employment, financial literacy, social skills, and household chores emerged as a shortcoming within conversations with individuals. Thus, implying that they believed the more emphasis placed on practicing these skills (e.g., writing resumes, asking/answering social questions, social skills), the more successful they would be in employment.

In addition to providing the opportunity for skill practice and generalization, practice at job sites had many positive outcomes. First, individuals, such as Emily, Ally, Tim, and John, were able to build relationships with community employers, which lead directly to employment after high school. Second, individuals had the opportunity to experience the nuances of employment, or as Tim said, “what working was like and different tasks that are done”. Third, individuals were able to find a job that fit with their preferences and characteristics, and employers were able to provide accommodations to meet their specific needs. As such, job site training provided an opportunity to acquire the skills for employment and practice those skills in the natural environment (Bouck, 2012; Carter et al., 2012).

Furthermore, in addition to learning and practicing skills directly related to job tasks and employment, unlike the conclusions of Bouck and colleagues (Bouck, 2012; Bouck & Joshi, 2015), participants expressed the importance of skills commonly found in functional curricula and IDEAL components, such as learning household chores and pre-employment skills (Bouck, 2012; Bouck & Joshi, 2015; Chaing et al., 2017) in both the survey and interviews. Specifically, the emphasis on life skills is a direct connection to IDEAL’s individualized programming and long-term outcomes. These include instruction of life skills and pre-employment skills, which are the areas participants felt were most beneficial instead of academic topics. It is possible that the difference here and in prior studies is related to the characteristics of individuals in this study as many prior studies queried a range of individuals. This seems to imply that studies giving voice to subsamples of autistic individuals (e.g., those immediately employed after high school, those who enrolled in postsecondary education, those with specific comorbidities) are needed to inform the field about specific needs in life skills instruction.

Several individuals perceived inappropriate student placement to be problematic and detrimental to their progress because of mistreatment, emotional issues stemming from inappropriate placement, and/or differences in functioning levels and abilities which acted as barriers in skill acquisition. Placement was obviously critical in creating a positive learning environment that promotes self-esteem, confidence, and therefore, independence.

Cumulatively, these findings, directly from autistic individuals who have engaged in K-12 life skills instruction and post-secondary employment, contribute support for the provision of multiple opportunities in the community to practice skills learned and needed to develop independence, decrease frustration in the workplace, and increase employability for some autistic individuals. We are honored to be able to share the voices of these autistic individuals with the community.

## Implications

### Implications for Practice

Implications specific to practice emerged. We encourage district level administrators, such as superintendents, to ensure special education programs are structured in a way that considers transition goals, potential post-secondary goals and outcomes, and integrates explicit life skills instruction, including pre-vocational and vocational skills, and job site training into programs for autistic individuals when indicated. When appropriate, curriculum should be evaluated so that it includes more elements of functional curriculum as it was reported as the most beneficial aspects of K-12 instruction as it related to immediate post-secondary employment for this subsample of autistic individuals. We also encourage principals and supervisors to carefully consider scheduling and student placements. Additionally, when appropriate, budgeting and community partnerships should be established so that autistic individuals have opportunities to engage in job site training as well as community-based instruction.

### Implications for Future Research

We strongly encourage researchers to include the voices of varying subsamples of autistic individuals in their work. Future research would benefit from continued study to elicit a range of lived experiences across the spectrum in relation to employment outcomes so that results can be generalized. Furthermore, the field would benefit from a longitudinal approach giving voice to autistic individuals with a variety of needs beginning in high school and during employment. Much more work is needed.

### Limitations

This study is not without limitations. The limitations are primarily related to the small slightly diverse sample who participated in the survey and interviews. The sample included limited variation in participants, including mode of communication as all participants communicated via spoken language. Additionally, individuals were mainly white, thus limiting the perspectives of autistic individuals from different racial and ethnic backgrounds. Another limitation is that socio-economic status was unknown. It is important to note that these findings cannot be generalized because of the limited sample size that comprises a small subset of the entire autistic population, therefore they are considered preliminary findings. Future research would benefit from including a variety of racial and ethnic backgrounds as well as information regarding socio-economic status to increase generalizability of results. Furthermore, half of the participants received their education in self-contained settings, which might indicate comorbid cognitive disabilities in some participants. A methodological limitation is that interrater reliability was not calculated for coding the qualitative data. It is possible that this combination influenced the findings. Although data from the survey were deemed reliable and in-depth information was gleaned from the interviews, results should be considered preliminary.

## Conclusion

As the prevalence rate and heterogeneity of ASD continues to steadily increase, planning for an equitable education for autistic individuals across the spectrum is essential to promote positive post-secondary outcomes. Findings from the current study and prior literature suggest that developing life skills, including vocational skills, is likely to be important for independence and employment outcomes, which might be achieved through job site placements and opportunities to engage in practice of specific skills, including job specific skills (e.g., folding clothes, creating spreadsheets), personal hygiene, household chores, pre-employment skills, and social skills. Our findings indicate potential benefits of including IDEAL components in K-12 life skills instruction as they are likely to relate to independence and successful employment outcomes. Most importantly, this study gave voice to a subsample of autistic individuals who expressed their experiences in K-12 life skills education as it related to employment status.

## Footnotes

1 All names of individuals, schools, and businesses are pseudonyms.
